# Current Status of Pediatric Labeling in China and the near Future Efforts Needed for the Country

**DOI:** 10.3389/fped.2014.00017

**Published:** 2014-03-26

**Authors:** Zhiping Li, Yi Wang, Dan Wu, Xuan Gao, Zhiyun Wang

**Affiliations:** ^1^Department of Pharmacy, Children’s Hospital of Fudan University, Shanghai, China; ^2^Department of Pediatrics, Children’s Hospital of Fudan University, Shanghai, China; ^3^Key Laboratory of Pharmaceutical Science, School of Pharmacy, Fudan University, Shanghai, China

**Keywords:** pediatric, label, information, integrity, China

## Abstract

**Background:** Children are recognized as “therapeutic orphan” in many parts of the world, one expression of this is the lack of adequate pediatric labeling information. Some research studies have been done to investigate the pediatric labeling condition in the U.S. and other countries, but no national studies had been carried out in China. This survey was conducted aiming to inquire the current situation of pediatric labeling in China.

**Methods:** We investigated 6020 child-applied medicines from 15 representative Chinese hospitals, and analyzed the information according to the dosage forms, therapeutic category, and label information integrity.

**Results:** Among all these medicines, only 238 (3.95%) are pediatric products, the rest are adult formulations with an extended use in children. The major pediatric formulations were injection (45.95%), tablet (23.69%), and capsule (4.93%), respectively. Alimentary tract/metabolism medicine (24.70%) and infections medicines (20.60%) had the most species. In prescription drugs, only 210 of 5187 (4%) medicines had adequate pediatric labeling information. The main cause of this deficiency was lack of evidence derived from pediatric clinical trials.

**Conclusion:** The dilemma of “therapeutic orphan” requires significant attention. Inadequate labeling information and lack of pediatric clinical trials were two prominent issues in China. It calls for more efforts from pharmaceutical industries, regulatory agencies, and legislature in China to collaborate and find solution to improve the situation.

## Introduction

Forty years ago, after the tragedy of fetal malformation from maternal ingestion of thalidomide, Dr. Harry Shirkey first pointed out the dilemma of pediatric therapy and raised the concept of “therapeutic orphan” (1, 2). That started the first step of the long journey of labeling all the therapeutic drugs for children. A complete label should include important information on indications and usage, clinical pharmacology, contraindications, precautions, adverse effects, dosage, and administration. Labeling of a drug indicates that there is adequate evidence from clinical trials to ensure the drug has reached an applied moral, ethical, and medical standard, so that it is safe and effective to use toward a certain population (3, 4). But pediatric labels often fail to reach that standard.

In 1975, Wilson investigated the US Physicians’ Desk Reference (PDR) and discovered only 22% of drug labeling had adequate pediatric information (5). In 2009, the proportion upgrades to 41% (6). China is the home to nearly 300 million children. The phenomenon of lack of labeling information in children is also a critical issue in China, yet very few studies have been carried out nationwide (7). This study was undertaken aiming to investigate the current pediatric medicine labeling situation with the intension that it may help to promote the collaborative efforts among pharmaceutical industry, the regulatory agencies of the country, and legislature professionals to provide child-friendly formulations and adequately label the medicines for infants and children.

## Materials and Methods

This study investigated the pediatric labeling in 15 top-ranked Chinese medical facilities by questionnaire, including the basic condition of pediatric drugs in each facility, their dosage forms, therapeutic category, and label information integrity.

### Basic condition

From July 2011 to December 2011, 15 Chinese medical facilities participated in the survey; they are either children’s hospitals or general ones that are top ranked in China and have excellent pediatric departments. Demarcated from national administrative zones, six were from Eastern China, including Children’s Hospital of Fudan University, Shanghai Xinhua Hospital, Nanjing Children’s Hospital, Zhejiang University affiliated Children’s Hospital, Anhui Provincial Children’s Hospital, Children’s Hospital of Harbin City; three from Central China, Beijing Children’s Hospital, Peking University Third Hospital, Children’s Hospital of Shanxi Province; five from the west, Chongqing Medical University affiliated Children’s Hospital, West China Women’s and Children’s Hospital, Children’s Hospital of Xian City, Children’s Hospital of Guiyang, Qinghai Women and Children Hospital, Guangxi Maternal and Children Health Hospital. These hospitals are representative in both the regional coverage and health care quality level. The results are reliable.

We investigated the labeling information for drugs used in children among the aforesaid hospitals. Drugs were divided into prescription and over-the-counter (OTC) products. Each category was then split into child-specific and child-used. Child-specific means medicines or formulations designed and produced to use exclusively in children. For example, poractant alfa (Curosurf) is the medicine used only in premature infants to treat respiratory distress syndrome. Ibuprofen suspension drops (Motrin) is the special formulation designed for children. Child-used definited as medicines or formulations designed and produced mainly for adults, but also have an extended use in children. For example, meropenem is used for both adults and children. The same compound or composition with different formulations, specifications, and pharmaceutical companies was double-checked to identify discrepancies on their indications and labeling integrity.

### Dosage forms

The dosage forms and the corresponding data and percentage were listed. The forms were classified as parenteral administration, oral administration, cutaneous administration, preparation for eye, ear, and nose, orally inhaled (pulmonary) medicine, and rectal administration.

### Therapeutic category

The therapeutic category and percentage of pediatric prescription and non-prescription medicines were investigated and analyzed. The anatomical therapeutic chemical classification system (ATC) was used.

### Label information integrity

The pediatric label information integrity of prescription medicines and non-prescription ones were shown in the following.

## Results

### Formulations intended for child-specific versus child-used

In all the investigated hospitals, there was a total of 6020 products used for children, 5187 prescription and 833 non-prescription drugs; while the child-specific were 124 (2.39%) among prescription drugs and 114 (13.69%) among non-prescription ones, respectively (Table 1). The child-specific proportion is low, and the phenomenon of adult products off-label use in pediatric population is serious in China, especially in prescription drugs.

**Table 1:** **Pediatric medication usage in selected hospitals**.

Hospital	Prescription drugs		OTC drugs	
	Child-specific	Child-used	Child-specific	Child-used
CHF	9	410	13	77
SXH	6	151	4	18
NCH	14	467	10	62
ZCH	13	467	11	61
ACH	7	334	7	49
CHH	10	266	9	30
BCH	10	508	13	54
PTH	6	482	13	117
CHS	12	340	3	44
CCH	9	371	7	49
WWC	7	481	7	68
CHX	10	288	7	34
CHG	6	139	5	18
QWC	5	253	5	19
GMC	0	106	0	19
Total	124	5063	114	719

*CHF, Children’s hospital of Fudan University; SXH, Shanghai Xinhua hospital; NCH, Nanjing children’s hospital; ZCH, Zhejiang University affiliated children’s hospital; ACH, Anhui provincial children’s hospital; CHH, the children’s hospital of Harbin city; BCH, Beijing children’s hospital; PTH, Peking University third hospital; CHS, the children’s hospital of Shanxi province; CCH, Chongqing Medical University affiliated children’s hospital; WWC, West China women’s and children’s hospital; CHX, the children’s hospital of Xian city; CHG, the children’s hospital of Guiyang; QWC, Qinghai women and children hospital; GMC, Guangxi maternal and children health hospital*.

### Dosage forms

In the survey, there were 32 dosage forms for child-specific and among them, injection (53.48%), tablet (21.62%), and capsule (4.81%) had the most species of dosage forms for prescription drugs; while tablet (36.75%), oral solution (9.31%), and ointment (8.35%) were common dosage forms for OTC drugs. The child-specific drugs covered 13 different dosage forms in prescription medicines and 11 forms in OTC ones, which both of them were less than half of child-used ones (Table 2).

**Table 2:** **Dosage forms of pediatric medicines**.

Dosage form	Prescription drugs		OTC drugs		Total
	Child-specific (%)	Child-used (%)	Child-specific (%)	Child-used (%)	
**PARENTERAL ADMINISTRATION**					
Injection	45 (1.63)	2721 (98.37)	0 (0.00)	0 (0.00)	2766
**ORAL ADMINISTRATION**					
Tablet	8 (0.56)	1110 (77.84)	19 (1.33)	289 (20.27)	1426
Capsule	9 (3.03)	240 (80.81)	1 (0.34)	47 (15.82)	297
Oral solution	19 (6.93)	150 (54.74)	39 (14.23)	66 (24.09)	274
Granule	25 (13.66)	99 (54.10)	16 (8.74)	43 (23.50)	183
Suspension	1 (0.70)	107 (75.35)	17 (11.97)	17 (11.97)	142
Syrup	1 (1.89)	22 (41.51)	6 (11.32)	24 (45.28)	53
Gelata	0 (0.00)	36 (78.26)	0 (0.00)	10 (21.74)	46
Dripping pill^a^	0 (0.00)	6 (27.27)	3 (13.64)	13 (59.09)	22
Emulsion	3 (16.67)	15 (83.33)	0 (0.00)	0 (0.00)	18
Elixir	6 (66.67)	3 (33.33)	0 (0.00)	0 (0.00)	9
Orally freeze-dried powder	0 (0.00)	8 (100.00)	0 (0.00)	0 (0.00)	8
Others^b^	1 (14.29)	2 (28.57)	1 (14.29)	3 (42.86)	7
**CUTANEOUS ADMINISTRATION**					
Ointment	0 (0.00)	95 (57.58)	0 (0.00)	70 (42.42)	165
Pulvis	2 (2.17)	47 (51.09)	7 (7.61)	36 (39.13)	92
Lotion	0 (0.00)	4 (14.81)	0 (0.00)	23 (85.19)	27
Liniment	0 (0.00)	11 (73.33)	0 (0.00)	4 (26.67)	15
Patch	0 (0.00)	8 (57.14)	0 (0.00)	6 (42.86)	14
Gargle	0 (0.00)	2 (28.57)	0 (0.00)	5 (71.43)	7
**PREPARATION FOR EYE, EAR, AND NOSE**					
Orphanology solution	0 (0.00)	145 (90.06)	0 (0.00)	16 (9.94)	161
Ophthalmology ointment	0 (0.00)	13 (61.90)	0 (0.00)	8 (38.10)	21
Ear drop	0 (0.00)	4 (80.00)	0 (0.00)	1 (20.00)	5
Nasal drop	0 (0.00)	2 (66.67)	0 (0.00)	1 (33.33)	3
**INHALATION (PULMONARY) MEDICINE**					
Aerosol(MDI)	0 (0.00)	132 (98.51)	0 (0.00)	2 (1.49)	134
Nasal spray	0 (0.00)	48 (80.00)	0 (0.00)	12 (20.00)	60
**RECTAL ADMINISTRATION**					
Suppository	4 (11.43)	13 (37.14)	4 (11.43)	14 (40.00)	35
Enema	0 (0.00)	5 (25.00)	0 (0.00)	15 (75.00)	20

*^a^ Dripping pill: softgel capsule containing medication that can be squizzed out for oral administration*.

*^b^ Others: including tincture, pills, and sugar pills*.

Noticeably, injection is the largest species in Chinese pediatric prescription drugs, but only 1 out of 60 (45/2766) injections were developed for pediatric use. The well majority of the injections, which children have access to were intended for adults, and most of them had no evidence from clinical trials to ensure the efficacy in pediatric population. Meanwhile, this may also raise the risk of therapeutic safety or even cause therapeutic disasters from either the pharmaceutical active component itself or the excipients. Benzyl alcohol, a bacteriostatic agent, is a common excipient in many intravenous products for adults, including cotrimoxazole, digoxin, phenobarbital, etc. It is likely to cause kernicterus, intra-ventricular, or “grasping syndrome” in pre-term infants due to their immature liver detoxification function (8, 9). In addition, it is worth questioning if pediatric patients really need so many injections.

Tablet and capsule were another two formulations and frequently chosen in children. When drugs for adults were extemporized into pediatric use, especially in infants and young children, the products need sub-divided, crushing, or sometime even dispersing, since young infants need less dosage or some children are unable to swallow the whole solid unit dosage forms. That unit tablet and capsule would result in a decreased dosing flexibility as compared to oral liquid preparations and oral flexible solid dosage forms (9). So more pediatric use formulations adapt to child-size and child administration route are greatly encouraged.

### Therapeutic category

According to WHO ATC-coding, alimentary tract and metabolism medicine (24.70%), Infections and infestations medicines (20.60%), and Brain and nervous system medicines (10.83%) were the main therapeutic category of Chinese pediatric drugs (Table 3).

**Table 3:** **Therapeutic category of pediatric medicines**.

Therapeutic category	Prescription drugs		Non-prescription drugs		Total
	Child-specific (%)	Child-used (%)	Child-specific (%)	Child-used (%)	
A	66 (4.44)	1044 (70.21)	69 (4.64)	308 (20.71)	1487
J	5 (0.40)	1215 (97.99)	0 (0.00)	20 (1.61)	1240
N	2 (0.31)	636 (97.55)	0 (0.00)	14 (2.14)	652
R	25 (4.92)	360 (70.87)	2 (0.39)	121 (23.82)	508
C	6 (1.45)	407 (98.55)	0 (0.00)	0 (0.00)	413
L	3 (0.73)	408 (99.27)	0 (0.00)	0 (0.00)	411
B	1 (0.34)	259 (89.31)	0 (0.00)	30 (10.34)	290
S	5 (1.92)	219 (83.91)	0 (0.00)	37 (14.17)	261
M	10 (4.95)	85 (42.08)	41 (20.30)	66 (32.67)	202
D	0 (0.00)	111 (56.63)	0 (0.00)	85 (43.37)	196
G	0 (0.00)	85 (100.00)	0 (0.00)	0 (0.00)	85
V	1 (0.36)	207 (75.27)	0 (0.00)	67 (24.37)	275

*A: alimentary tract and metabolism; J: infections and infestations; N: brain and nervous system; R: respiratory system; C: cardiovascular system; L: malignant and immune disease; B: blood and blood forming organs; S: sensory organs; M: muscles, bones and joints; D: skin; G: genitourinary system; V: others, including Diagnostic agents, Disinfectants and antiseptics, Radioactive drugs*.

Infections and infestations medicines had the most species in pediatric prescription drugs. But, there is only one type of pediatric specialty formulation. As a result, the adult antibiotics and adults formulations are commonly used in children that may cause potential risk especially in neonates and small infants. Besides antibacterial, nutrition and NSAIDs are the most widely used drugs in pediatric population, which showed the consistency with several European studies (10, 11).

### Label information integrity

The integrity of prescription and non-prescription medicines was shown in the following tables (Tables 4 and 5). We analyzed the items in the drug instructions, and compared condition of child-specific and child-used ones.

**Table 4:** **Pediatric label information integrity of prescription medicine**.

Drug information	Child-specific (number)	Child- specific (%)	Child-used (number)	Child- used (%)
Dosage	120	96.77	2562	50.60
ADR	86	69.35	603	11.91
Contraindications	71	57.26	371	7.33
Special notes	90	72.58	885	17.48
Indications	94	75.81	4089	80.76
Drug interactions	64	51.61	132	2.61
Pharmacokinetics	56	45.16	628	12.40
Drug overdose	57	45.97	301	5.95
Clinical trials	12	9.68	198	3.91
PD/toxicology	76	61.29	225	4.44

*ADR, adverse reaction; PD, pharmacodynamics*.

**Table 5:** **Pediatric label information integrity of non-prescription medicine**.

Drug information	Child-specific (number)	Child- specific (%)	Child-used (number)	Child- used (%)
Dosage	110	96.49	334	46.45
ADR	60	52.63	30	4.17
Contraindications	50	43.86	43	5.98
Special notes	99	86.84	604	84.01
Drug interactions	51	44.74	16	2.23

*ADR, adverse reaction*.

There were fewer items in drug instructions for pediatric OTC medicines.

Among all the drug instruction items, the integrity of “indication” and “dosage” was much higher than the rest. That may be understood as drugs available must first focus on the essential need for pediatric use. Given that “clinical trials” had got the least description in the prescription medicine and was omitted in most OTC package insert, drug selection, and dose titration for children have to be based on clinical researches issued on medical literatures or extrapolation of adult labeling information. That would likely cause ineffective therapy or toxic results in pediatric patients.

In general, child-specific medications showed higher integrity on label information than child-used, for both prescription and non-prescription medicines. As such, if it is optional, child-specific medicines are strongly recommended. They not only possess better labeling integrity, but also more considerable for child use, for example masking the odd smell, making medicines child-size.

## Discussions

### Therapeutic orphan in China

Although the concept of “therapeutic orphan” was raised more than 40 years ago, the therapeutic dilemma had not been significantly changed. The phenomenon of extrapolating dosing information from adults to children is still in many areas worldwide. Yet, we clearly recognized that the effects of many drugs on children may vary dramatically from what was seen in adults. The main reason is significant physical and maturation changes that occur from newborn to adolescence. Ontogeny of drug absorption, distribution, metabolism, and elimination makes children different from adults (12, 13).

In the United States, there were several milestones in the pediatric labeling process. As early as the late 1930s, after 107 children death cause by diethylene glycol administration, the food, drug, and cosmetic act (FDCA) was amended to include not only a requirement to provide adequate directions for use and disclosure of composition and method of preparation of medicines, but also evidence of products’ safety (14, 15). Another well-known tragedy worldwide was induced by thalidomide, which entered the market in 1957 as a sedation to alleviate nausea and vomit during pregnancy, unfortunately, the gestation mother were “vulnerable populations” to this new drug, and more than 10,000 cases of birth defect over 30 countries were caused. As a response, the Kefauver–Harris Drug Amendments was made in 1962. The thalidomide issue also reminds people that the safety and efficacy in ordinary adults cannot be applied to vulnerable populations (16–18). So in 1979, the Food and Drug Administration (FDA) required that recommendations for pediatric use should be based on evidences from adequate and well controlled studies in pediatric population, unless they were waived. This revision was intended to encourage pediatric clinical trials, but turned out to be in opposite effect. The 1994 Final Rule for pediatric labeling continued to clarify the FDA position on various procedures that can get pediatric labeling, and compromise that in certain cases, drugs may be labeled for pediatric use if there were adequate evidence from well controlled studies in adults, with pharmacokinetic and safety data in pediatric population. What’s more, from 2002 to 2003, the Best Pharmaceuticals for Children Act, in exchange for study the drug in children, the drug maker gets more 6 months of selling their products without competition, and the Pediatric Research Equity Act gives FDA the right to ask drug companies to study the effectiveness of new drugs in children (19).

The teratogenic effect of thalidomide also strengthened the awareness of the European authorities. As a result, Directive 65/65/EEC1 was installed. EU law is having a considerable effect on drug development in children. The new legislation [European Regulation (EC) No. 1901/2006], named the “Pediatric Regulation” governing the development and authorization for use of medicines in the child population, entered into force on 26 January 2007 (20). The Pediatric Regulation brings in many new tasks and responsibilities for the European Medicines Agency (EMA). One of the most important is the creation and operation of a Paediatric Committee (PDCO) within the EMA (21). The Committee includes pediatric experts from all over the EU. The main responsibility of the PDCO is to assess the content of pediatric investigation plans (PIPs) that are proposed by industry, and to provide opinions on them in accordance with the Pediatric Regulation. For new medicinal products of therapeutic interest for children, the EU requires early submission of a PIP for approval by the PDCO. PIP submissions are expected to be filed at the time of completion of adult clinical pharmacology and pharmacokinetic studies (phase I), i.e., early in product development, in order to facilitate dialog and avoid any delay at the time of marketing authorization application. The objective is to ensure that medicines intended for eventual use in children are evaluated and authorized appropriately in all pediatric age groups, including neonates.

In China, the lack of evidence supporting drug use in children is also causing many serious problems. Yang investigated the special education schools in China, and found nearly 70% of the deformity children were caused by irrational use of medicines (22). Nevertheless, there are still very few regulations and policy legislated to improve the safety and efficacy use of medicines and clinical trials for Chinese children. In 2003, the State Food and Drug Administration (SFDA) published Good Clinical Practice, and regulated if a child was joining in the clinical trial, informed consent must be signed by their guardian (23). In 2004, SFDA published Guidelines of Vaccine Clinical Trials, mentioned that when clinical trials is conducted in children, there must be adequate consideration on ethical issues (24). But the good intention to protect children may actually have opposite effect, since conducting clinical trials in children means extra cost and risk confronted by pharmaceutical companies.

The result of our study shows that only 210 out of 5187 child-applied prescriptive medicines had information derived from clinical trials, even some of them only possess evidence in adult clinical trials and pharmacokinetic and safety data in children. While in non-prescription medicines, the data in pediatric clinical trials is nearly blank.

### The issue of China’s pediatric labeling integrity

From the study methods established by Wilson (3, 5) and his followers (6), labeling was categorized as adequate if it stated that the drug was approved for pediatric use, had been studied, and had safety, efficacy, dosing information for all appropriate pediatric populations, which we adapted as indication, clinical trials, adverse reaction, and dosages in different groups in our study.

In Wilson’s study, there was only 22% of all prescription electronic Physician’s Desk Reference (ePDR) had pediatric information in 1975, the ratio raised to 41% in 1999 and 46% in 2009. In 2009, 50% of prescription child-applied medicines were adequately labeled for pediatric use. Topicals, nasal sprays, and most OTC products were excluded per the methods of Wilson.

In our study, we evaluated 5187 child-applied prescription medicines, among which only 4% products had adequate pediatric information, which is far behind the ratio of U.S. The main reason is that only a few drugs had been carried out clinical trials in pediatric population. If the medicines were re-evaluated when “clinical trial” item was removed, the adequate label rate was 13%.

The label integrity standard between Chinese prescription and OCTs are quite different. In prescription drugs, dosage, adverse reaction, contraindication, special notes, indication, drug interaction, pharmacokinetics, drug overdose, clinical trials, and PD/toxicology are included, while in OTCs, only dosage, adverse reaction, contraindication, special notes, and drug interaction are involved. Within the categories, different medicines shared different integrity. In general, the essential medicines from international drug manufactories usually have better integrity than domestic generic drugs. To unify the level of pediatric label integrity, national legislation must intervene.

Many of unlicensed and off-label drugs are not available in pediatric formulation and had to be modified by the pharmacy department to make them suitable for administration to children. Stability data are rarely available for such products, which are rendered unlicensed by this modification.

Another issue is the generic drugs. Generic drugs are very common in China, different regions may have different medical enterprises producing the same drug, but the levels vary greatly, and the integrity of labels is also different. The hospitals in different regions prefer, using their local products besides the imported ones, since they are easier to get. Yet this may cause not only the label integrity problems, but also serious health risks for the receivers (patients).

### Developing pediatric specialty drug products in China to satisfy the unmet medical need

In December 2007, WHO launched an initiative for “Make Medicine Children Size” in order to encourage and accelerate research, development, and test of appropriate dosage forms for pediatric population. This action highlighted the urgent need for accessibility and availability of pediatric formulations. Challenges for appropriate pediatric dosage forms are not only derived from the unique status of physiological conditions and disease states, but also the heterogeneous nature of pediatric patients. For example, children groups are sub-divided as (1) new born (0–27 days); (2) infant (28 days–2 years); (3) toddlers (2–5 years); (4) children (6–11 years); adolescents (>12 years old). Each age group has different preference to the dosage forms and therefore, should drive the decision of pediatric dosage form selections. For example, injectable medication is preferable for the new born. Syrup, oral solution or suspensions are mostly appropriate for the infants and toddlers. Studies conducted in Norway showed that more than 50% of children prefer liquids/suspensions even for 12 years old. Younger children, especially at disease stage, strongly prefer liquids/suspensions (25, 26). Age appropriate specialty formulations in pediatric population should consider the following factors: (1) Safety: excipients, drug degradation products, potential interactions between excipients and drug substance are proven safe and acceptable to the targeted population group; (2) Easy administration and dosing accuracy: this is particularly important for medications that will be given based on body weight or surface areas. The dosage form should allow flexibility in accurately sub-dividing the dose, such as measured by volume for oral solution or suspensions; (3) palatable: children are very sensitive to the taste. Many medications have un-pleasant taste, and thus should be properly taste-masked in pediatric dosage forms to ensure compliance and adherence. Adherence to the full strength dose and dosing regimen are the most important measures to achieve therapeutic efficacy as well as to reduce the side effect; (4) appropriate dosing device: this is to ensure accurate delivery of appropriate doses; but unfortunately is a largely ignored area.

In summary, accessibility of appropriate pediatric drug products to pediatrician, hospitals is the critical element in “providing right medicine to children.” Appropriate pediatric dosage forms will provide a powerful tool for clinicians to conduct pediatric clinical trials that lead to better pediatric labeling; and most importantly allow accurate delivery of medications to children.

## Conclusion

Children, the “therapeutic orphan,” seem to be more marooned in China. Despite the inadequate labeling information and fewer clinical trials, there are even more problems for this group in China, such as imbalanced ability to access the essential or better medicines in different regions, the duplication of generic drug at a low level, and the parents’ blindly trusting in the use of injections. In the developed countries, people began to realize the special characteristics and need of children more than 50 years ago, a series of law and regulations had been formulated to increase pediatric labeling situation. But in China, there is no national list of pediatric essential medicines, and there is very little pharmaceutical legislation to protect children from harms of irrational drug use from the national level.

In the twenty-first century, China is developing rapidly in many aspects, but as far as we are really willing to get some benefit to our children, and make pediatric drug use safer and more effective, we need to work together since China is the home of nearly 300 million children. The pharmaceutical industry need to sponsor the necessary studies, the SFDA enforce its regulations, the legislature to mandate all child-applied medicines should be evaluated appropriately, and the public be adequately educated, if all of these may be transformed, the therapeutic orphan will disappear into an awkward moment of medical history.

## Conflict of Interest Statement

The authors declare that the research was conducted in the absence of any commercial or financial relationships that could be construed as a potential conflict of interest.
